# Probabilistic regression for autonomous terrain relative navigation via multi-modal feature learning

**DOI:** 10.1038/s41598-024-81377-z

**Published:** 2024-12-02

**Authors:** Ickbum Kim, Sandeep Singh

**Affiliations:** Mechanical, Aerospace, and Nuclear Engineering, Rensselaer Polytechnic Institute, 110 8th St, Troy, 12180 NY USA

**Keywords:** Convolutional Neural Network (CNN), Cascading architecture, Machine Learning (ML), Image processing, Astronomy and planetary science, Engineering, Computer science, Software

## Abstract

The extension of human spaceflight across an ever-expanding domain, in conjunction with intricate mission architectures demands a paradigm shift in autonomous navigation algorithms, especially for the powered descent phase of planetary landing. Deep learning architectures have previously been explored to perform low-dimensional localization with limited success. Due to the expectations regarding novel algorithms in the context of real missions, the proposed approaches must be rigorously evaluated in extraneous scenarios and demonstrate sufficient robustness. In the current work, a novel formulation is proposed to train CNN-based Deep Learning (DL) models in a multi-layer cascading architecture and utilize the resulting classification probabilities as regression weights to estimate the position of the lander spacecraft. The approach leverages image intensity and depth data provided by multiple sensors on board to accurately determine the spacecraft’s location relative to the observed terrain at a specific altitude. Navigation performance is validated through Monte Carlo analysis, demonstrating the efficacy of the proposed DL architecture and the subsequent state-estimation framework across several simulated scenarios. It shows tremendous promise in extending the multi-modal feature learning approach to realistic missions.

## Introduction

The next generation of space missions will be focused on demonstrating success by taking incremental steps with the ultimate aim of establishing humans as a multi-planet species. The short-term efforts will be focused on sustaining human presence on our nearest neighbor in the cosmos, the Moon^1,2^. A variety of mission architectures have been explored in this context, which typically include sending manned spacecraft to a periodic orbit (an orbit from the Near Rectilinear Halo Orbit (NRHO) family) around the Earth-Moon Lagrange Point ($L_2$)^3,4^, then maneuvering the spacecraft to a Low-Lunar Orbit (LLO)^5,6^ and finally detaching the lander from the orbiter and eventually descending onto the lunar surface, making a soft landing on the lunar terrain. NASA’s Commercial Lunar Payload Services (CLPS)^7,8^ is a high-risk, high-reward approach for performing key science missions and technology demonstration that would be instrumental in enabling a sustained human presence. Recent efforts by commercial participants like Astrobotic’s Peregrine lander^9^ and Intuitive Machines’ IM-1 mission^10^ have paved the way for a high volume of ‘first-timers’ attempting to fly spacecraft in the cislunar domain and eventually perform a soft landing close to the Lunar South Pole. A successful soft-landing sequence has several design and operational challenges. In particular, due to the geometry of the Earth-Moon system and the lunar south pole, communication between the spacecraft and the Earth-based ground station suffers from signal attenuation and in extreme scenarios may result in complete signal blockage due to a lack of line-of-sight. Therefore, autonomy is paramount to enable real-time decision-making during the spacecraft’s descent from orbit. Planetary landers are generally equipped with sensors that provide input to the onboard computer which estimates the current state of the spacecraft - both position and attitude. The objective of a subset of the onboard sensors is to perform Hazard Relative Navigation (HRN)^11^ whereby the spacecraft avoids hazardous terrain while descending and Terrain Relative Navigation (TRN) which enables localization of the spacecraft in the absence of Earth-based Global Navigation Satellite Systems (GNSS) relative to the observed planetary terrain.

TRN has emerged as an integral component of autonomous planetary landing, with optical sensor inputs utilized to estimate the current state of a spacecraft relative to known features on the planetary terrain. Integrating Machine Learning (ML) frameworks has been transformative in developing autonomous processes. In the context of TRN, training of deep learning models can be performed from data prepared from planetary reconnaissance missions, such as the Lunar Reconnaissance Orbiter (LRO), which captured the lunar surface at a resolution of approximately one meter per pixel^12^ or the Mars Reconnaissance Orbiter (MRO)^13^. This deep-learning approach relies on accurate and robust feature-matching as the key cornerstone for success. Feature matching algorithms that localize craters and other landmarks against known databases based only on optical inputs i.e., images experience extreme sensitivity to changes in illumination conditions, camera angle, and other numerical errors^14,15^. Landmark selection algorithms have been somewhat helpful in diminishing these undesirable effects^16^. On the other hand, Visual Odometry (VO) paradigms which do not require extensive cataloging for TRN have also majorly advanced the capabilities of autonomous systems and vastly improved robustness to the prevalent sources of error^17,18^. However, for real-time space applications, VO tends to be less efficient due to the computational complexity and budgeted on-board computational overheads.

Optical and laser-based sensors have become increasingly accurate in the past couple of decades and enable reconstruction of the three-dimensional surroundings. LiDAR and stereo camera pairs are now commonly included in the sensor suite of planetary landing modules. Depth information from a LiDAR and a stereo-pair has been demonstrated to complement each other and has been effectively used to upsample depth information while improving the accuracy of the assimilated 3D point-cloud as shown in^19^. Real and simulated LiDAR data has proven efficient and robust data for neural network training in many applications, such as obstacle detection^20^, path planning^21^, and TRN even in the case of uncooperative operations^22^. Stereo vision was the main component of the navigation sensor-suite used to execute TRN in the Mars 2020 Lander Vision System^23,24^. Stereo vision in combination with IMU inertial data has been previously used to localize the descending lander module to land with an accuracy on the order of ten meters^24^. Previous works have trained Convolutional Neural Network (CNN)-based classifiers that classify images taken from the lander’s perspective into one of several classes in 1D position sub-space, as shown in^25^. Another work used a Siamese Neural Network architecture capable of performing image matching and results in a position retrieval system for reliable autonomous navigation^26^. Other works have explored the use of Reinforcement Learning (RL) for autonomous planetary landing^27,28^.

As mentioned before, posing the TRN problem as a ‘classification-only’ problem results in challenges in model training and the approach is generally not scalable. It was shown in^25^ that the model performance even for a simple 1D classification was $\approx$ 25 % which does not meet the demands of the Technology Readiness Level (TRL) expected for real space missions. The cascading architecture developed in^29^ simplified the complex learning task by reducing the number of classes and posed the problem as a ‘quadrant-classification’ problem instead, improving on the performance for 2D classification. However, the test accuracy from this approach across several illumination conditions was $\approx$ 60–70%, with poor performance in low-illumination conditions. In the current paper, a novel TRN model architecture is proposed which is trained on multi-modal data i.e., both intensity from the 2D images and the 3D depth map obtained as a result of the onboard stereo-pair. For this work, it is assumed that the spacecraft (lander) is retrofitted with a stereo-camera pair and takes nadir-pointed images during the descent phase. The onboard computer converts the stereo images to a depth map and this information is leveraged to localize the spacecraft relative to the observed terrain. Additionally, a probabilistic regression approach is used to evaluate the best estimate of the spacecraft position, relative to the terrain. With the help of simulated scenarios, it is conclusively demonstrated that the inferior performance, especially in low-illumination conditions of the classification-only cascading CNN model, can be significantly improved by leveraging features from both modalities during training. The proposed model— (1) improves test accuracy, (2) leverages classification probabilities to localize the spacecraft, and (3) improves robustness and generalizability during realistic mission scenarios. The rest of the paper is organized as follows. Firstly, the problem is formulated, outlining the specific research questions and objectives addressed in this study. Next, the methodology section explains our approaches to dataset generation for training and test, including preprocessing, and augmentation techniques employed to enhance the dataset’s quality and diversity. Subsequently, the architecture of the Multimodal cascading CNN is discussed. Following this, the results obtained from the experimentation and evaluation of the proposed methodology are presented and analyzed, with case studies where the TRN performance is evaluated using the results of the probabilistic regression. To evaluate uncertainty and quantify navigation performance, a 2500-run Monte Carlo analysis was conducted, providing insights into the model’s reliability. Finally, the conclusion section summarizes the main contributions of the research, discusses its implications, and offers recommendations for future work in the field.

## Problem formulation

TRN provides critical information for the successful implementation of a planetary soft-landing sequence. It is imperative to locate the descending lander relative to the designated landing area to provide inputs to the onboard computer and compute a control profile that ultimately results in a soft landing. Due to the time delay associated with any Earth-based communication, the state information must be computed autonomously to serve as input for any onboard guidance algorithms, which require fast response time. Any delays or inaccurate estimations can result in catastrophic failure as observed in several legacy missions to the Moon.

**Fig. 1:**
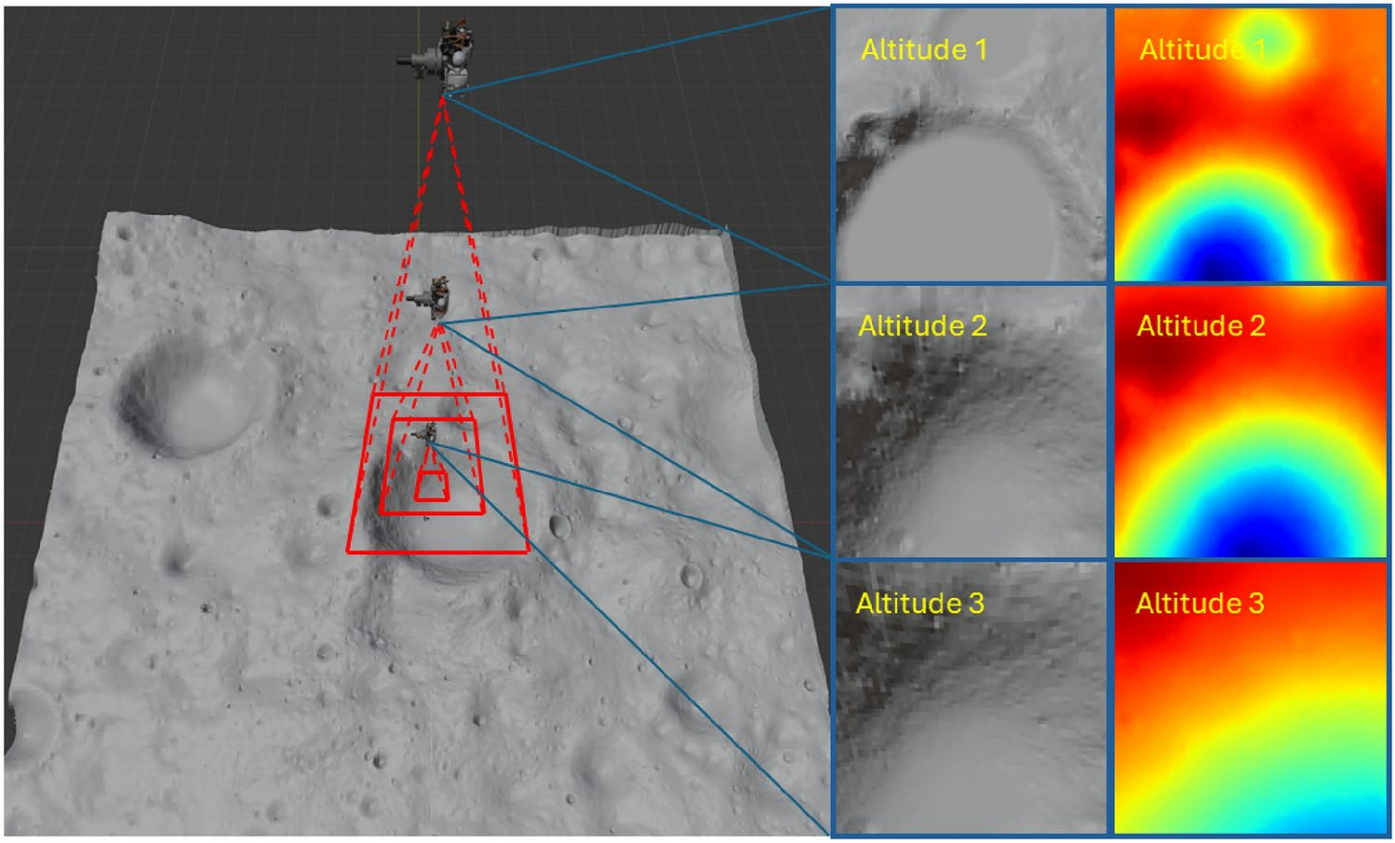
Terrain relative navigation overview.

Consider a lunar lander, equipped with a stereo-camera pair among other sensors, intending to land on the surface of the Moon. The lander begins its descent from a pre-identified selenocentric orbit. Note that, typically before the descent sequence is initiated, the lander’s attitude is corrected in a controlled fashion such that the cameras take near nadir-pointed images during descent. It is then desired to perform autonomous TRN leveraging pre-trained models uploaded to the onboard computer. Figure 1 depicts the descent sequence and image acquisition through controlled spacecraft pointing. For this work, it is assumed that the autonomous TRN is initiated at a pre-defined altitude (gathered by the altimeter) above the surface, denoted by *h*. At this altitude, the TRN problem can be defined as a 2-dimensional position estimation problem. With known stereo-camera parameters, the current lander position ($\varvec{\hat{X}}$) is estimated using the center of the image acquired at the current altitude ($\varvec{\hat{x}} = \{\hat{u},\hat{v}\}$) and an atlas ($\mathbb {S}$) spanning a bigger lunar terrain area which includes identified target landing site ($\mathbb {L}$). Given that $\mathbb {L}$ and $\varvec{\hat{x}}$ can be located in $\mathbb {S}$ at all ‘*N*’ discrete altitude levels where TRN is sought, a feedback controller can be implemented between the *i* *th* and $(i-1){th}$ levels $\ni h_{i-1} > h_{i} \ \forall \ i = 1,2,\ldots N$ until a successful soft landing is achieved within a certain tolerance, i.e., $h_N = 0$. It should be noted that the solution to the described TRN problem should display robustness under realistic conditions that may lie on the boundary of the design sub-space. For instance, although the lander descent would ideally be carried out under sufficiently bright illumination conditions, lunar craters (especially around the south pole) are permanently shadowed. Therefore, any autonomous TRN approach should also demonstrate acceptable performance under partially visible or low-illumination conditions.

## Methodology

The proposed methodology is based on a trained model that learns features from a multi-modal dataset. It is hypothesized that training a model to make predictions based on multi-modal data will demonstrate robustness even in extreme operating conditions while also improving performance under nominal conditions due to the additional feature knowledge. This hypothesis is based on the heuristic insight that intensity variation in images with significant shadowed regions is minimal, and therefore result in ambiguous performance if trained only on intensity maps or images. This can be compensated by supplementing the initial dataset with more features, which is provided by a depth map in the current work. Additionally, a cascading neural network architecture is proposed to reduce the dimensionality of the initial classification problem, making it numerically and computationally more tractable by reducing the complexity of feature interactions at each stage and partitioning the overall task into smaller, more manageable subproblems. This approach enables the model to progressively filter and narrow down potential classes, thereby focusing computational resources on fewer, more relevant categories as it proceeds through each cascade layer. A probabilistic regression scheme leveraging cumulative classification probability densities across the cascading layers is proposed instead, and shown to demonstrate consistent performance over the entire domain ($\mathbb {S}$). The key aspects are discussed in more detail in subsequent sections.

### Overview

The Digital Terrain Model (DTM) for the Apollo 16 landing site was acquired from the Lunar Reconnaissance Orbiter Camera website^30^ and serves as the source model for dataset extraction. The DTM has embedded depth information which can be supplemented with a simulated light source, leading to a synthetic generation of an intensity map by computing radiant intensity from the surfaces. With the incorporation of depth information as one of the training inputs in the cascading CNN, the model becomes adept at capturing intricate relationships. By harnessing both depth and intensity data, the CNN can extract rich features, thereby enabling robust categorization of the lander’s location into quadrants while also quantifying the corresponding prediction confidence levels. Moreover, these prediction confidences across different quadrants serve as inputs for transitioning to a regression model, thereby facilitating a more precise position estimation.

### Dataset generation

**Fig. 2:**
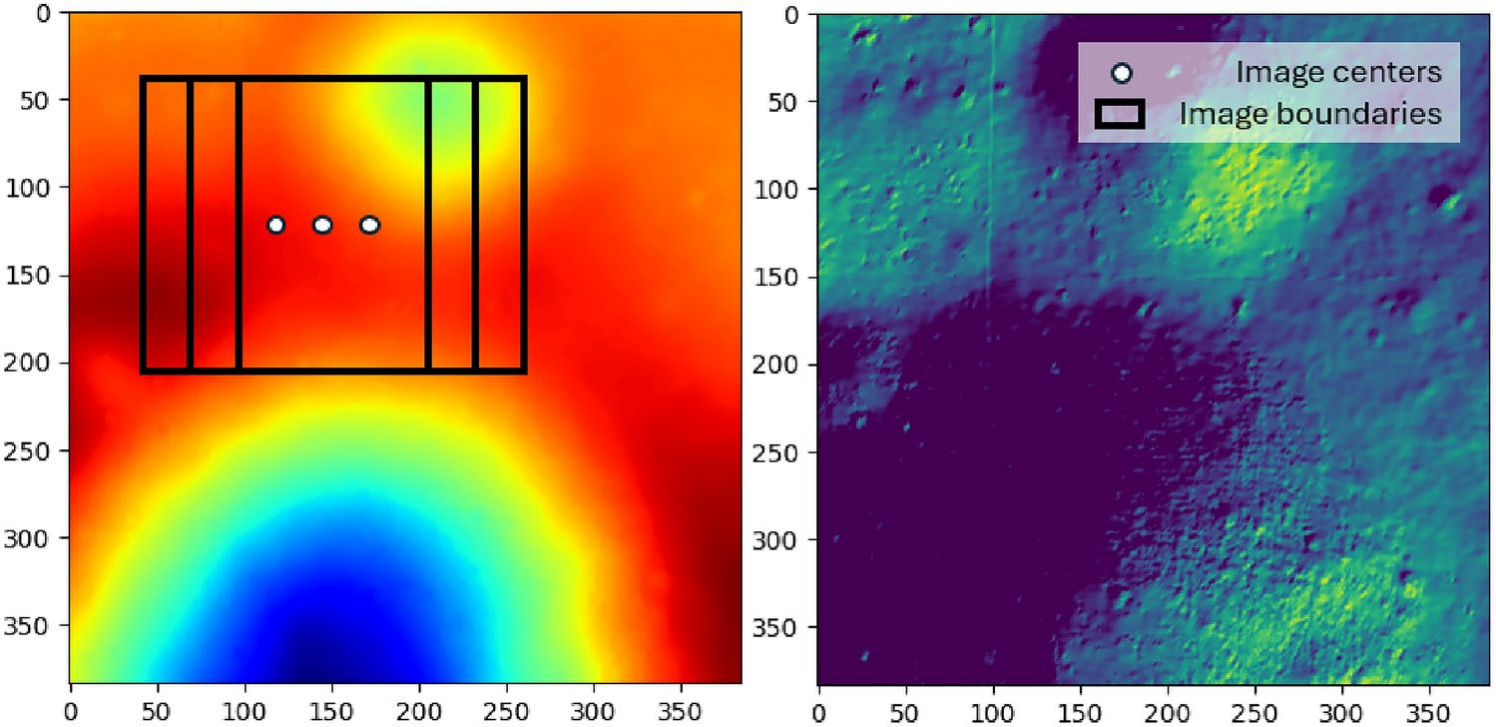
Dataset generation example. (Left) Depth map with visualization of image centers and boundaries. (Right) Intensity map.

This section offers a comprehensive explanation of the methodologies employed to generate the training and test datasets. As mentioned earlier, the proposed architecture utilizes two features of the available data: depth and intensity. Both the depth and intensity datasets are generated based on a lunar descent scenario, where nadir-pointed images are captured by a descending lander at an altitude 352 meters above the lunar surface. Camera calibration is assumed to have been completed prior to the mission, so no additional image processing is required. The resolution of the image segments is determined through the following trigonometric relation:

1 $$\begin{aligned} px = \frac{2h\tan \bigg (\frac{\text {AOV}}{2}\bigg )}{R_{px}}, \end{aligned}$$

where *px* denotes the window size, $R_{px}$ is the available DTM resolution $(2 \text {m/pixel})$, *h* is the imaging altitude, and AOV signifies the angle of view of the camera, chosen as 40°  for this work. Due to the relatively low resolution of the DTM, the window size for the training sub-sampled images is set to 32, 64, and 128 pixels respectively, for three cascading levels. These specific resolutions are chosen due to their power-of-2 nature, facilitating convenient sub-division. Figure 2 depicts the approach for dataset generation. It is hereby noted that the approach is scalable and robust in that when a higher resolution DTM is available, models can be efficiently trained for lower altitudes. Therefore, for the current discussion the raw DTM resolution presents a bottleneck.

#### Depth dataset

A $384 \times 384$ area is selected to accommodate the necessary $64 \times 64$ padding, which prevents data loss when the image centers are positioned near the landscape boundaries. The selection of the landscape is conducted carefully to ensure that the minimum simulated camera height does not pass below the maximum height depicted in the Lunar DTM. Image center locations are sampled between pixels 64 and 320 in both the x and y directions, then $128\times 128$ images are cropped and down-sampled to $32\times 32$. Subsequently, the $64\times 64$ and $32\times 32$ images are sampled similarly. However, subdividing into smaller quadrants diminishes the number of images falling within the sub-category, necessitating denser sampling locations. In this study, sampling locations are spaced with increments of 2, 4, and 8 for window sizes 128, 64, and 32, respectively to generate an equivalent number of images for each sub-category. Each sampled image is assigned a label between 1 to 4 based on the quadrant where the image center resides.

For rigorous performance evaluation of the trained model, test datasets are created by sampling depth images of equal resolution at image center locations chosen randomly between 64 and 320. Both the training and test depth datasets are also augmented with Gaussian noise, with the noise level for test being three times more significant than that utilized for training, to simulate a more realistic evaluation of performance.

#### Intensity dataset

The information from images captured during descent can be simulated in the presence of different illumination conditions to build the dataset. Lambert’s cosine law serves as the foundation for generating the intensity map from the depth information. This law asserts that the radiant intensity observed from a Lambertian surface is directly proportional to the cosine of the angle $\theta$ between the observer’s line of sight and the surface normal. However, since the dataset is constructed based on a hypothetical scenario where the lunar descent model is assumed to be positioned directly towards the center of the moon, the radiant intensity from the surface is computed as follows:

2 $$\begin{aligned} I_r = I_i cos(\theta ), \end{aligned}$$

where $I_r$, $I_i$ represent the radiant and incident intensity, and $\theta$ is the angle between the incident ray and the surface normal. Negative intensity values are adjusted to zero to signify shades. The surface normal for each point is determined based on the gradient in the x, y, and z directions, which are then normalized by dividing with the magnitude. Subsequently, 10 random combinations of azimuth and elevation angles within the specified ranges (outlined in Table 1) are selected to generate the intensity maps for further sampling.

**Fig. 3:**
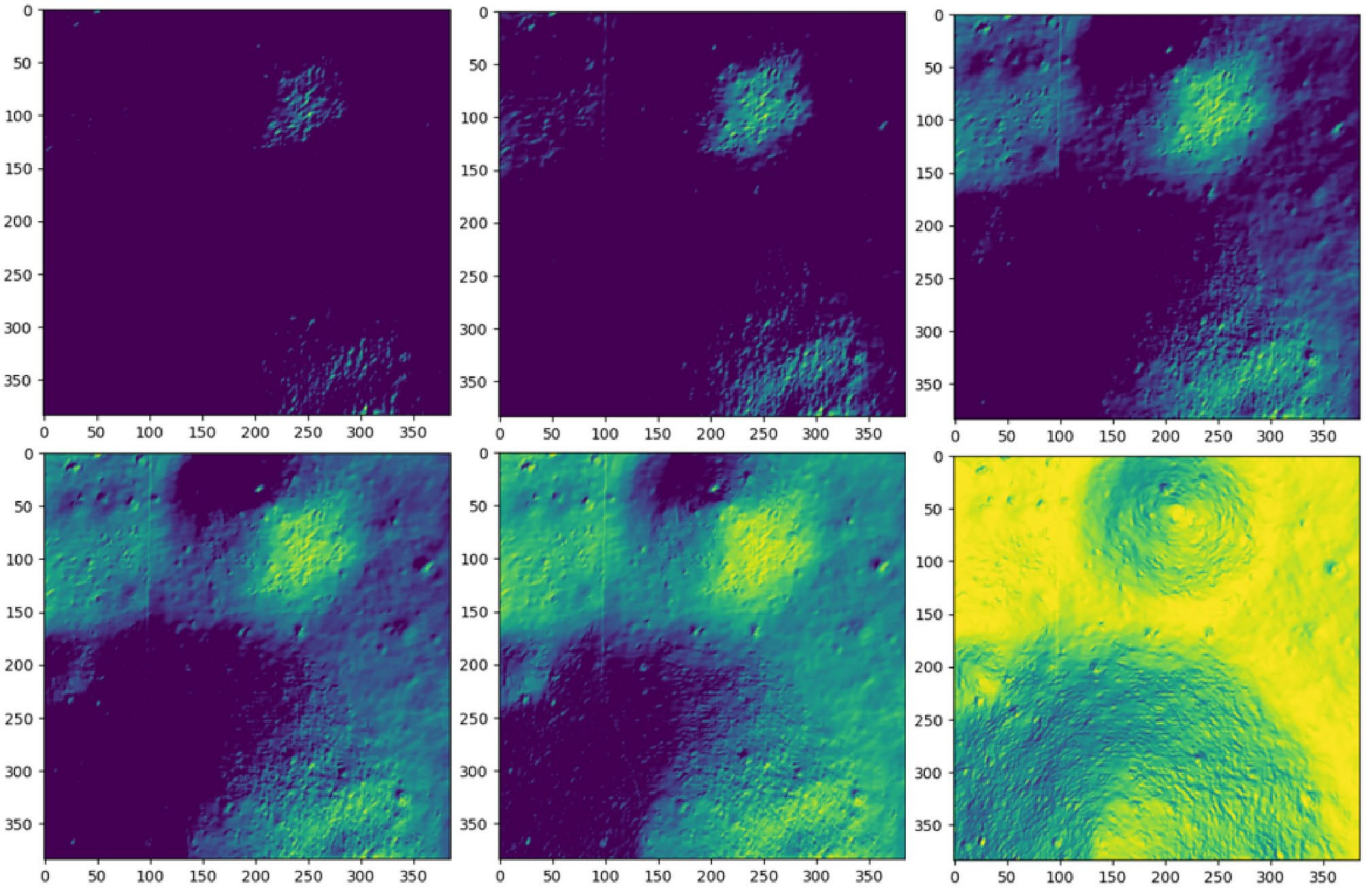
Image excerpts from the intensity dataset, in the increasing order of Sun elevation angle from left-top to right-bottom.

Once the intensity map is generated from the DTM, images are sampled using the approach outlined in previous section, maintaining identical resolutions and heights. A distinct test dataset is crafted to assess the model’s generalizability across varying illumination conditions and depth uncertainties. To create a realistic test dataset, intensity maps with authentic sun angles are generated. These angles are calculated with predetermined azimuth angles across 50 equally spaced elevation angles between 0 and 180 degrees, encompassing potential low illumination scenarios. Figure 3 illustrates examples of intensity maps generated from realistic sun angles utilized in creating the test dataset. Additionally, intensity maps with extremely low visibility are included in the test dataset, although they are not depicted in Fig. 3, for clarity. The realistic images are sampled from a random uniform selection of image centers between 64 and 320. Table 1 outlines the parameters employed in generating the training and test datasets.

**Table 1:** Dataset generation parameters.

Displacement	Training dataset	Test dataset
Lowest real-world elevation	− 107.7 m	− 107.7 m
Highest real-world elevation	41.3 m	41.3 m
DTM resolution	2 m/px	2 m/px
Lighting conditions		
Number of angles	10	50
Azimuth Angle	0–360°	45°
Elevation	0–180°	0–180°

#### Extreme test set

**Fig. 4:**
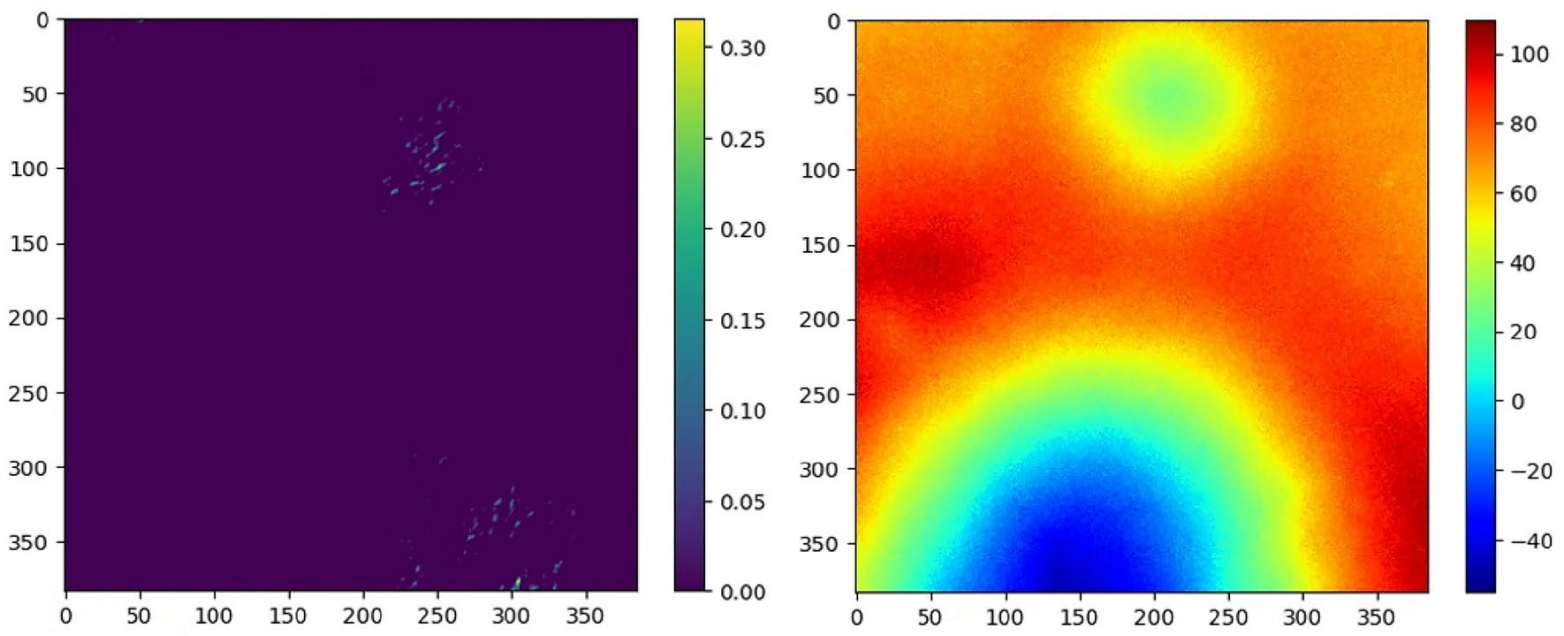
Extreme conditions example. (Left) Low illumination intensity. (Right) Noisy depth map.

To evaluate the model’s performance under extreme conditions characterized by severely contaminated or low-quality data or partially visible terrain, an extreme test dataset is prepared. This dataset simulates low illumination conditions for the intensity dataset by utilizing equally spaced incident rays with elevation angles ranging from 0 to 20 degrees to generate intensity images. Depth data is augmented with truncated Gaussian noise, with $(\mu ,\sigma ) = (60,10)$, to simulate scenarios where a high degree of sensor error is induced by factors such as changes in calibration parameters and inefficient stereo depth algorithms. Figure 4 (Left) illustrates the intensity map generated with an elevation angle of 20°, while (Right) depicts the corresponding noisy depth map over ($\mathbb {S}$).

### Multi-modal cascading CNN architecture

**Fig. 5:**
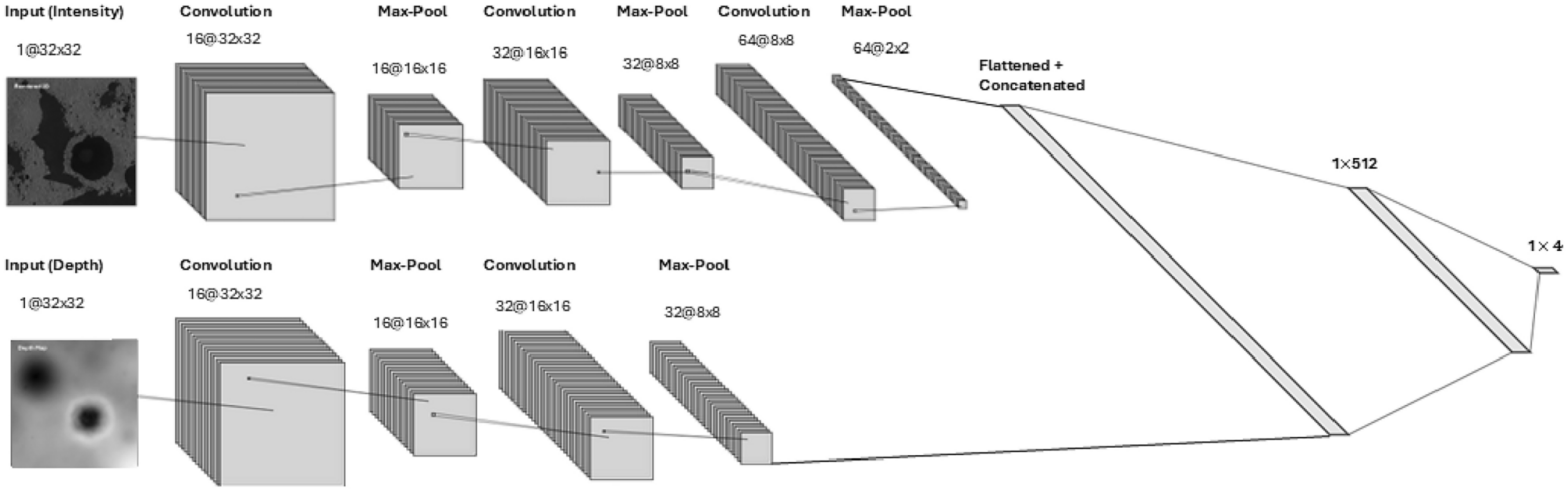
Architecture of the MMCNN.

To avoid over-fitting, promote regularization, and encourage sparsity during training, diversity within the depth training dataset is limited to a defined window of the scene. For instance, 128-pixel resolution depth images are generated with an 8-pixel increment, restricting the size of the training dataset to $(256/8)^2 = 1024$ samples. Conversely, while sampling locations for intensity are similarly confined, the intensity training dataset can be substantially augmented by introducing slight variations in the “sun angle”, thereby generating diverse intensity images within the same window. However, the generation of multiple intensity maps from sun angle variations inevitably leads to the repeated training of identical depth images when employing a uni-mode 2-channel CNN architecture. Consequently, an imbalance between the diversity of depth and intensity images introduces bias, compelling the model to emphasize features extracted from depth, more significantly, leading to a loss of sensitivity to intensity-based features. Common strategies to address this imbalance in different dataset channels include ensemble training, where multiple models are trained and combined to make system-level decisions, and multi-modal structure, where the architecture incorporates multiple modes for separate feature extraction. In this study, we adopt the multi-modal architecture to alleviate these concerns. Moreover, Gaussian noise is introduced to the depth dataset to augment variation, while a dropout rate of 0.5 is applied to supplement efforts to prevent overfitting. Figure 5 demonstrates the architecture of the Multi-Modal CNN (MMCNN).

A comprehensive list of the model hyperparameters is provided in Table 2. By definition, a dropout rate of 0.5 randomly selects half of the latent features and sets their outputs to zero. By dropping half of the data passed to the fully connected layers, this process helps prevent overfitting and significantly enhances the model’s adaptability to various datasets, including the extreme conditions discussed earlier.

**Table 2:** Cascading MMCNN hyperparameters.

Parameter	Value
Batch size	128
Epochs	500
Learning rate	0.01
Momentum	0.4
Dropout	0.5
Stride (convolutional layers)	1
Optimizer	Stochastic gradient descent (SGD)^31^
Loss	Negative log likelihood (NLL)

The MMCNN architecture is built upon two independent nodes, each calling for individual investigation. Discovering the relationships between intensity images proves more intricate, given the inclusion of more challenging data instances such as low-illumination images with only partially decipherable features. Consequently, the node dedicated to intensity requires greater “depth”. Establishing a symmetrical MMCNN architecture with equally deep nodes escalates computational cost during training while opting for shallower networks for both nodes compromises performance. Thus, the architecture for the two nodes is selected based on their respective performance characteristics. Following feature extraction, these features are flattened and concatenated. Subsequently, the concatenated features are passed into fully connected network layers to facilitate classification into a quadrant.

Multiple MMCNNs from respective image sample sizes are stacked to narrow down the location of the agent (spacecraft). For instance, three specific sub-sample levels lead to a categorization problem with 64 classes. Increasing the number of levels (*l*) will provide a higher dimension classification with $4^l$ categories. Additionally, layers(*n*) of the cascading architecture requires training of $\Sigma _{k = 0}^n 4^{k-1}$ models at each height level.

### Regression

**Fig. 6:**
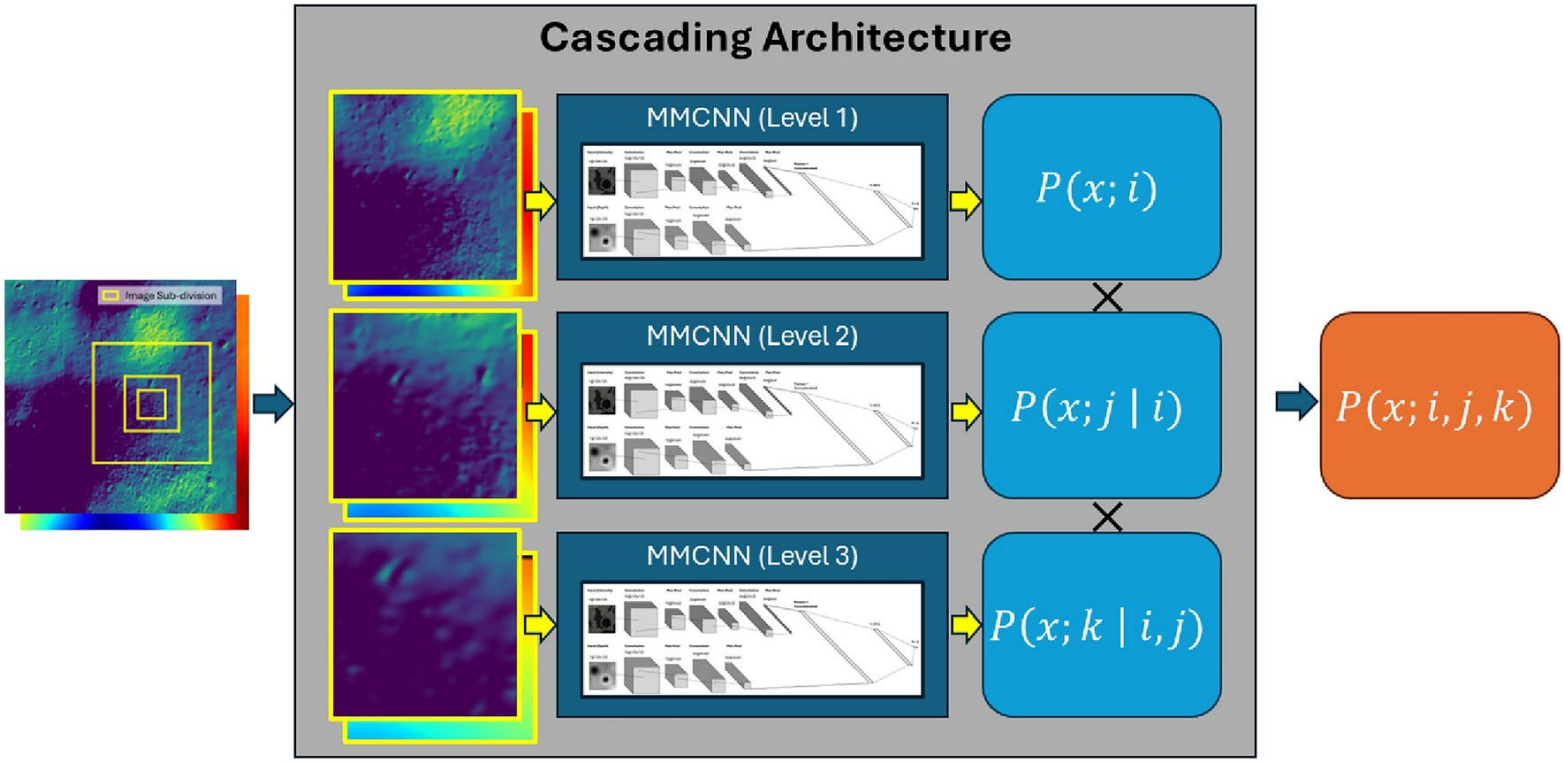
Joint prediction confidences.

The proposed MMCNN utilizes softmax for the categorization process. Softmax serves as an activation function, transforming arbitrary real-value scores into a probability distribution across multiple classes. Mathematically, the softmax function is defined as follows:

3 $$\begin{aligned} \sigma (z_j) = \frac{e^{z_j}}{\Sigma ^K_{k = 1}e^{z_k}}, \end{aligned}$$

where $\sigma (\cdot )$ represents the softmax function, $z_j$ denotes the input vector, and *k* stands for the number of classes. The output of the softmax function can be interpreted as the prediction confidence distributed across all potential classes. Thus, employing the cascading architecture yields a joint probability distribution that can be utilized for downstream regression. Suppose, $P(x;q \mid r)$ denotes the prediction of input *x* in quadrant *q* given *r*. The joint probability assimilating prediction confidences across the cascading architecture is then expressed as:

4 $$\begin{aligned} P(x;i,j,k) = P(x;i)P(x;j\mid i)P(x;k \mid i,j), \end{aligned}$$

where $i,j,k \in \{1, 2, 3, 4\}$. The calculation of joint probability is visualized using the schematic in Fig. 6. Due to the computational overhead of computing confidences, for the TRN application, it is important to identify a *goldilocks* cadence of estimating states, building a guidance algorithm, and controlling the lander.

Given *P*(*x*; *i*, *j*, *k*) and the corresponding image center ($C_{i,j,k}$) for the sub-sub-quadrants, the predicted location of the input image *x* can be formulated as follows:

5 $$\begin{aligned} (c,r) = \sum _{i,j,k = 1}^4 P(x;i,j,k)C_{i,j,k}, \end{aligned}$$

where *c* and *r* represent the column and row in the image, respectively. Two distinct approaches are employed, and subsequently compared: (1) Centered Prediction, and (2) Mean Neighborhood Prediction.

#### Centered prediction

**Fig. 7:**
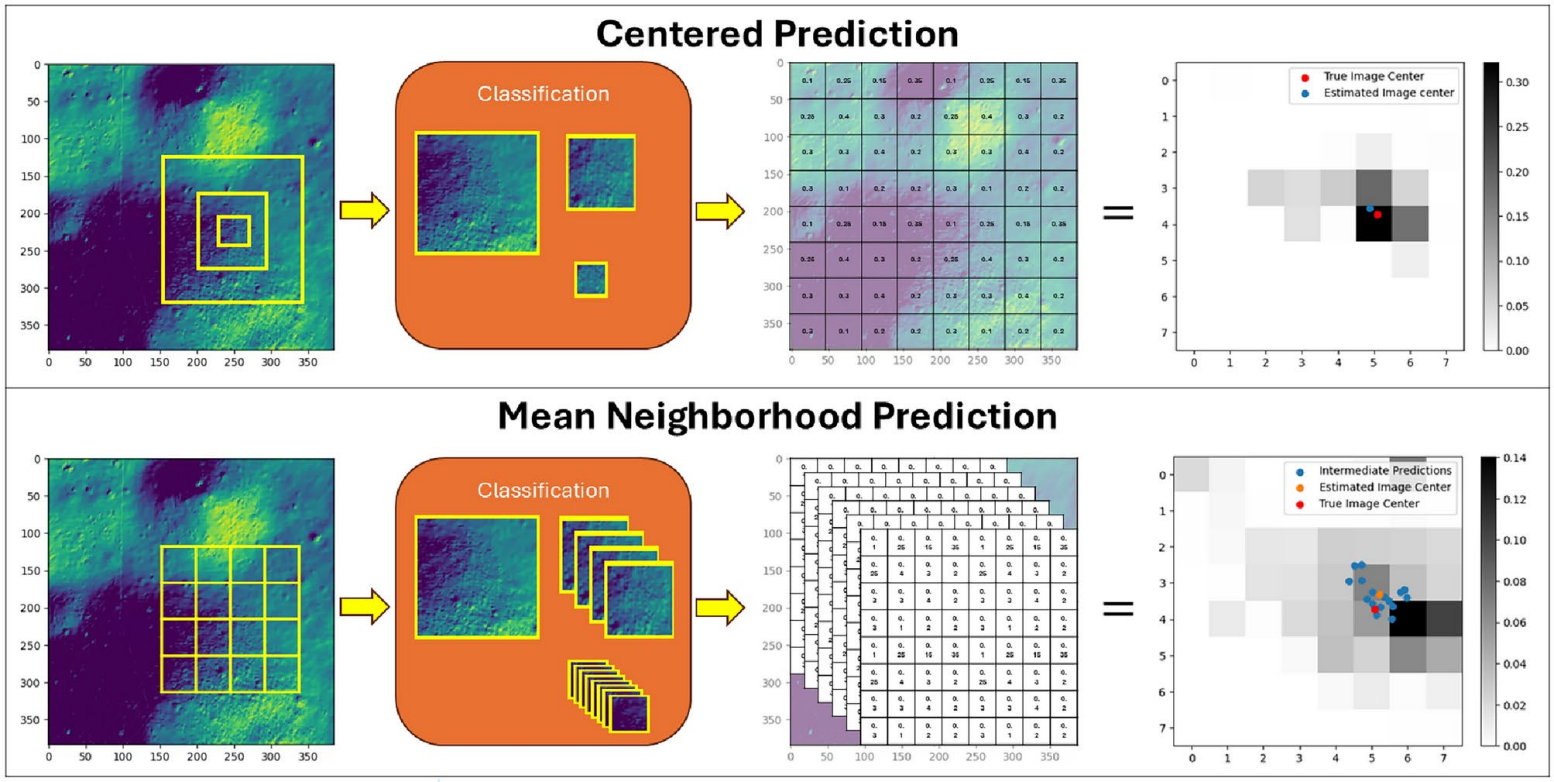
Centered prediction and mean neighborhood prediction comparison.

This approach is relatively straightforward and is based on combining information from all levels of the cascading architectures through cropped images while retaining the image center. For the described approach, three different segments are extracted from the input image i.e., the image captured by the lander during descent. It is assumed that the stereo pair images are also simultaneously captured and a stereo algorithm is employed to render a depth map. The $128\times 128$ segment undergoes classification using the model trained at level 1, followed by the $64\times 64$ segment, which is processed by four trained models at level 2. Subsequently, the $32\times 32$ segment is subdivided into 64 divisions using 16 trained models at level 3. Since the models at level 2 are subdivisions of those at level 1, and level 3 further subdivides level 2, the joint probability can be computed using Eq. (5) to generate a heatmap over the entire domain surrounding the intended landing site.

An illustrative example describing the centered approach is presented in Fig. 7, where the accumulative sum of grid probabilities equals 1. This is in contrast to the conventional Cascading CNN categorization proposed in^29^, where the estimated image center can fall anywhere within the window. The joint probabilistic regression approach offers a more precise prediction of the image center, thereby leading to a more accurate state estimation relative to the observed terrain.

#### Mean neighborhood prediction

While the *Centered Prediction* approach is simpler in formulation and has minimal computational overhead, it does have a notable drawback. As segments are extracted around the image center, a substantial amount of data is lost from the smaller window segments. Therefore, this leads to biased estimates based only on the classification probabilities of the three cropped images, leading to inaccuracies, especially, in the presence of noise and outliers in the current image dataset. To address this issue, a mean neighborhood approach is proposed where predictions are made on every subdivided segment within the image and a weighted sum based on the cumulative probability density map leads to an estimation of the image center. An example application of mean neighborhood prediction is illustrated in Fig. 7.

#### Temperature scaling

Trained networks for image classification tasks tend to suffer from overconfidence, which is not ideal and can skew the downstream regression towards biasness. For the examples discussed earlier, despite achieving an impressive accuracy on the test set, the model often exhibited a tendency to assign excessively high probabilities to its predictions, especially for challenging or ambiguous samples. This overconfidence becomes particularly problematic when deploying the model in real-world scenarios, where accurate estimation of the prediction uncertainty is crucial for future decision-making. Consequently, to address this issue and improve the reliability of the model’s confidence estimates, temperature scaling was implemented:

6 $$\begin{aligned} \sigma (\frac{z_i}{T}) = \frac{e^{\frac{z_i}{T}}}{\Sigma _j e^{\frac{z_j}{T}}}, \end{aligned}$$

where $z_i$ is the unnormalized logit for class *i*, *T* is the temperature scale, and $\sigma$ is the softmax function from Eq. (3). By applying temperature scaling to the logits (raw outputs) of the neural network, the model’s confidence is recalibrated to better reflect the true uncertainty of its predictions. This approach proved to be highly effective, as it not only mitigated the problem of overconfident predictions but also enhanced the regression task. Note that while there does exist an optimal Temperature scale (*T*) for a particular model, *T* was retained as a flexible hyperparameter for this paper.

## Results

To explore the robustness of the multi-modal CNN, two uni-modal CNNs were also constructed, each focusing on a respective modality (depth and intensity). These uni-modal CNNs were also separately evaluated on each of the test datasets to serve as the “control” for performance evaluation, as prepared and discussed in Section “Dataset generation”.

### Test results

The test results confirm the initial hypothesis with consistent performance at all levels. For instance, test result for Level 1 is shown in Table 3. The test accuracy for the MMCNN notably surpasses that of the intensity-only uni-modal CNN, increasing from 76.4 to 99.3%. Remarkably, the uni-modal CNN for depth achieves a high accuracy of 96.8%, despite being trained on a sparsely sampled dataset. This suggests that features extracted from intensity maps offer supplementary information to compensate for the limitations faced by the depth-only CNNs.

**Table 3:** Level 1 test result.

Modes	Test accuracy (%)
Intensity only	76.4
Depth only	96.8
Intensity + depth	99.3

Table 4 shows test accuracy comparisons at level 2. A similar trend is observed in the test results for Level 2. Despite exhibiting a low accuracy of 57.4% in the third quadrant, the MMCNN demonstrates consistent performance above 98%. Table 5 presents the test accuracy comparisons at Level 3. Despite fluctuations in accuracy for uni-modal CNNs, the MMCNN consistently maintains reliable accuracy above 93%. The higher accuracy for the depth CNN compared to the intensity CNN follows the expected pattern. This is because the depth datasets are generated directly from ground truth depth, with variations induced solely by the addition of a Gaussian noise. In contrast, the intensity datasets exhibit more complex relationships and variations due to factors such as varying sun angles, making it a more challenging task for the CNN to precisely model the intensity images.

**Table 4:** Level 2 test result.

Level 2 quadrant	Intensity (%)	Depth (%)	Intensity + depth (%)
1	74.00	96.18	98.80
2	77.00	96.25	98.99
3	57.40	97.60	98.70
4	77.29	96.36	98.62

**Table 5:** Level 3 test result.

Level 2 quadrant	Level 3 quadrant	Intensity (%)	Depth (%)	Intensity + depth (%)
1	1	68.88	90.19	97.42
	2	69.57	93.06	97.52
	3	64.38	96.21	98.31
	4	65.97	95.12	97.81
2	1	68.67	97.76	98.06
	2	78.04	96.27	97.65
	3	69.76	96.20	96.00
	4	61.47	43.79	93.31
3	1	57.04	91.89	96.96
	2	50.69	98.08	97.65
	3	52.20	96.09	96.29
	4	52.20	98.14	98.73
4	1	61.03	94.54	96.80
	2	70.44	89.46	93.56
	3	75.68	96.58	97.39
	4	77.53	84.35	95.29

**Fig. 8:**
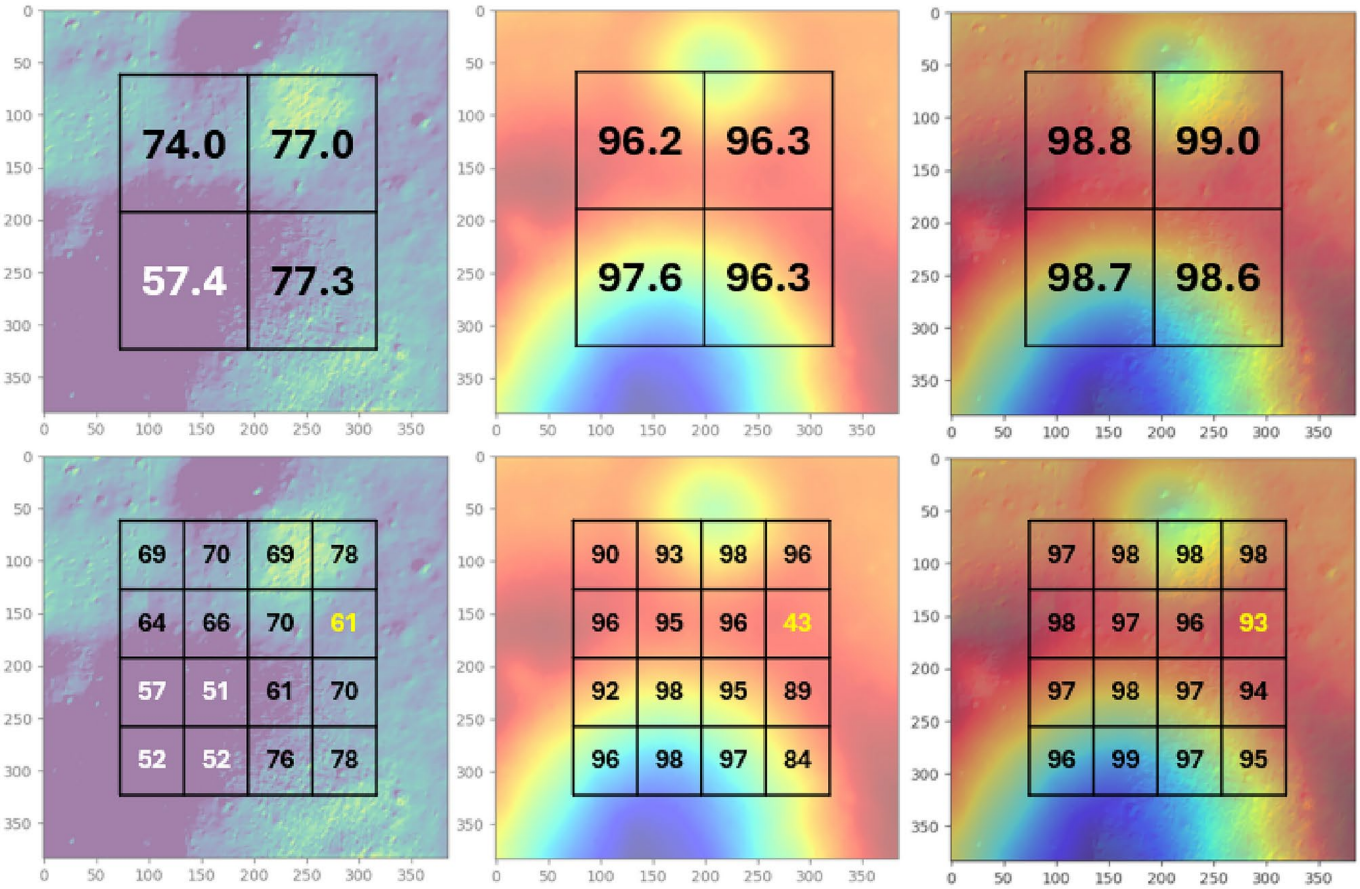
Visualization of Level 2, 3 results. (Top) Level 2. (Bottom) Level 3. (Left) Intensity map. (Middle) Depth map. (Right) Intensity and depth.

Figure 8 is included to provide visualization and further explanation of the relationship between the quadrant location and corresponding test accuracy. Quadrants and sub-quadrants with accuracies that significantly deviate from the others are highlighted in white numbers in Fig. 8. A distinct pattern emerges in the accuracy for intensity, where lower intensity regions correspond to lower accuracy. Conversely, the accuracy for depth exhibits a notable dip to 43% in quadrant 2 sub-quadrant 4. Upon analyzing the dataset used to train this specific sub-quadrant, it was discovered that the depth variation within the boundary is significantly lower compared to other sub-quadrants, resulting in insufficient or a lack of meaningful features. However, the combination of intensity (61%) and depth (43%) marked in yellow exhibits a notable synergy, resulting in a significantly higher accuracy of 93%.

### Extreme cases test

While the results presented in the previous section offer valuable insights, the test accuracy for single depth CNNs is already too high to discern any significant improvement in leveraging the multi-modal feature training over depth-only training. Furthermore, it is crucial not to underestimate the necessity of testing drastic cases to simulate the worst-case scenarios, particularly considering that missions are not always executed under ideal conditions and often require architectural redesign forcing it to demonstrate acceptable performance in severely off-nominal conditions. Therefore, additional tests are conducted on the extreme test set, wherein only low illumination conditions are selected for the intensity dataset and a high degree of noise is augmented in the depth dataset. Table 6 displays the corresponding test results.

**Table 6:** Severe environment test.

Modes	Intensity (%)	Depth (%)	Intensity + depth (%)
Low illumination	34.7	–	98.3
Noisy depth	–	52.3	65.3
Low illumination + noisy depth	34.7	52.3	65.0

It is immediately evident that the test accuracy decreases in both uni-mode CNNs, with 34.7% for intensity and 52.3% for depth. However, the MMCNN accuracy where low illumination conditions are supplemented by depth information, substantially improves performance to 98.3%. Similarly, the MMCNN accuracy for the extremely noisy depth map case (with regular intensity images) demonstrates a significant increase of 13%. Introducing extreme cases for both nodes results in a consistent positive accuracy of 65%. It is evident that the accuracy is more reliant on the features provided by the depth dataset, given the absence of depth data or corrupted depth data with errors caused by factors such as misalignment of the lunar descent sensor, changes in ground-based optical calibration during the several mission stages (predominantly launch), and severe space-weather conditions. However, the discussed results demonstrate that the performance of MMCNN and its utilization of multiple modes of data provide a clear improvement and enable meaningful interpretation, thus cannot be understated.

### Regression result

This section discusses the regression results for the Centered Prediction (CP) and Mean Neighborhood Prediction (MNP). Root Mean Squared Error (RMSE) between the prediction center $(\varvec{\hat{x}})$ and the ground truth $(\textbf{x})$ is calculated to evaluate the respective regression performances, and corresponding heatmaps are presented for visualization.

**Fig. 9:**
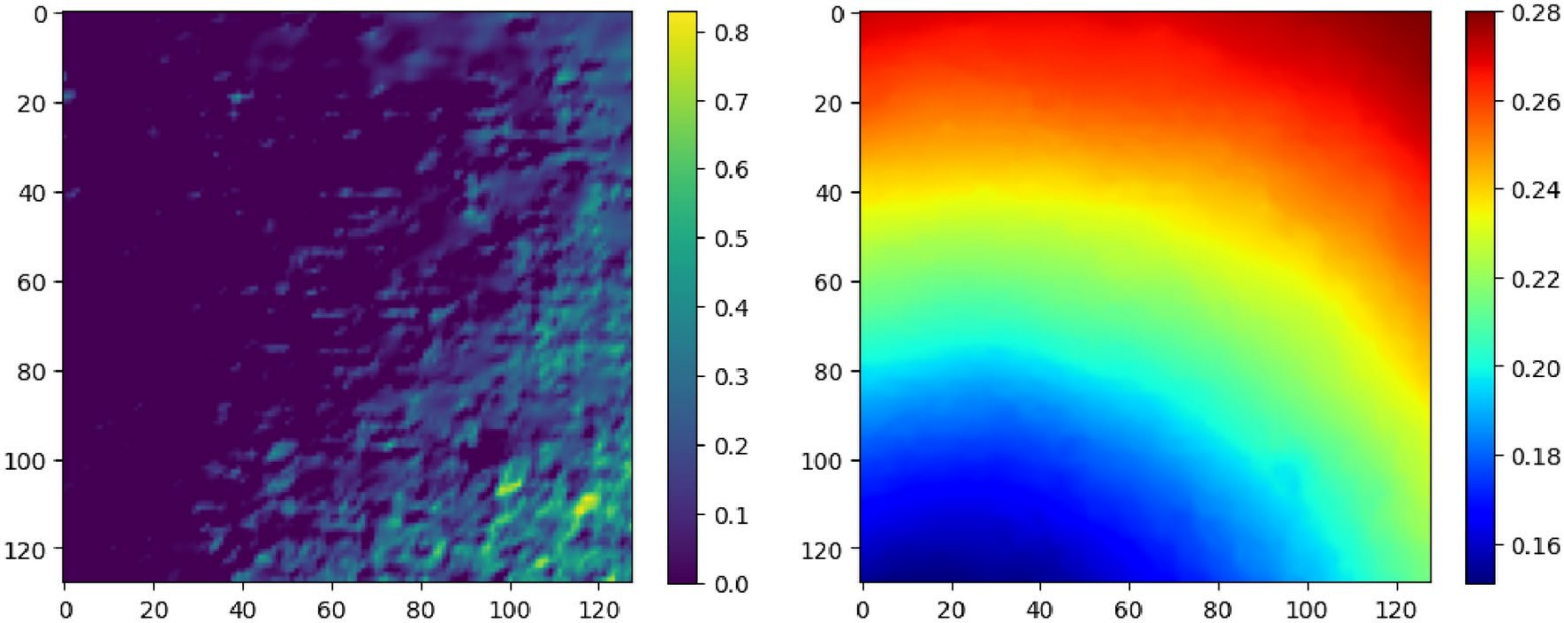
Regression Case 1 with image center at (200,250). (Left) Intensity image. (Right) Depth image.

**Fig. 10:**
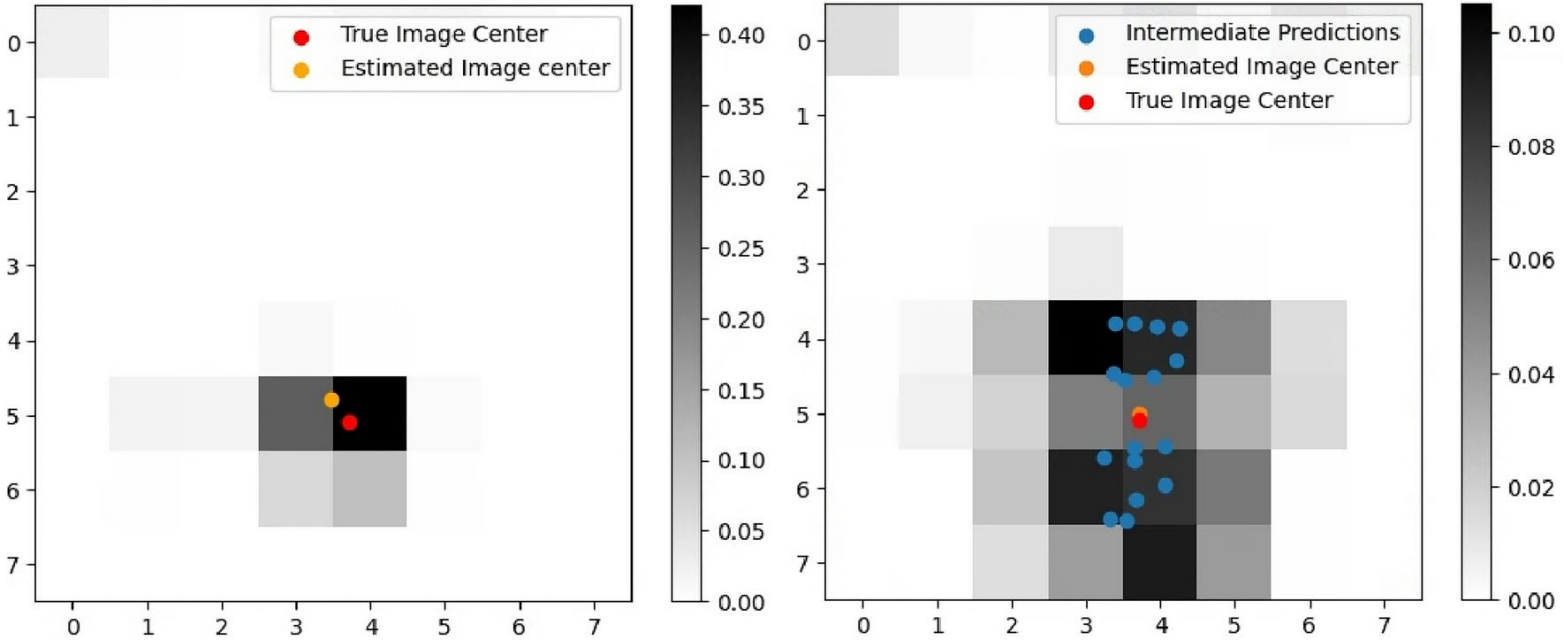
Heatmap of regression results. (Left) Centered prediction result with RMSE of 10.01. (Right) Mean neighborhood prediction result with RMSE of 1.98.

First regression case study was done using the images sampled at (*x*, *y*) = (200, 250) as shown in Fig. 9. The regression results for the selected image demonstrate promising outcomes for both methods, with accurately identified image centers. The depth and intensity images are generated using the same approach from the test dataset. The RMSE for CP and MNP are 10.01 and 1.98 pixels, respectively. Upon conversion to real units, this corresponds to a deviation of 20.02 and 3.96 meters. The heatmaps are depicted in Fig. 10, where the heatmap for MNP (on the right) is generated by averaging the heatmaps from 16 image subdivisions.

**Fig. 11:**
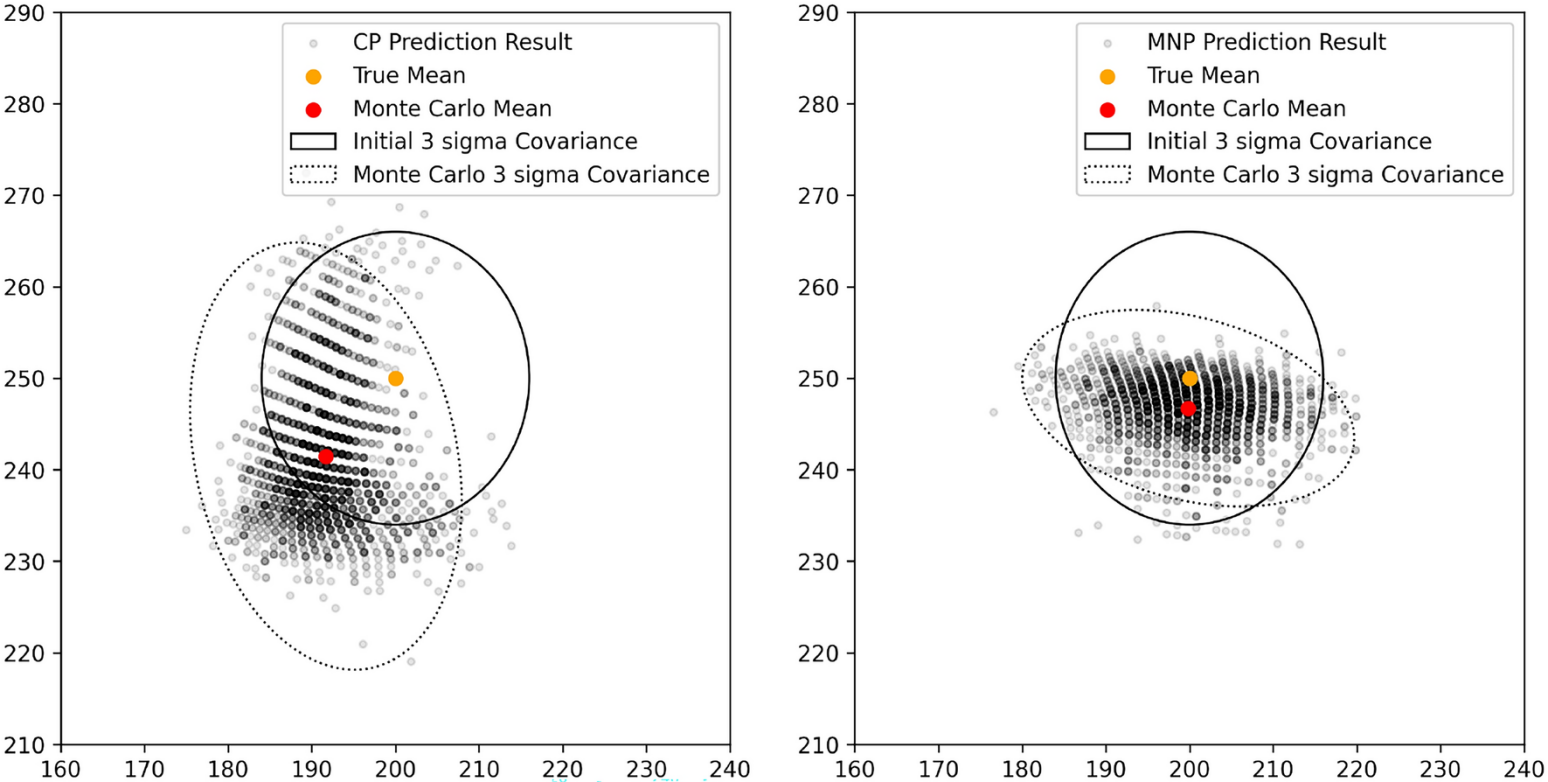
Visualization of 2500-run Monte Carlo Analysis with initial image center at (200, 250) and $3\sigma$ covariance bounds. (Left) Centered prediction result with mean (191.7, 241.5). (Right) Mean neighborhood prediction with mean (199.9, 246.7).

Monte Carlo (MC) analysis is performed to assess the uncertainty within the regression model, similar to the approach described in^32^. To achieve this, random pixel locations are generated using a bivariate Gaussian distribution, with image center coordinates (200, 250) from Fig. 10 as the mean, and a $3\sigma$ radius corresponding to 16 pixels. This radius is chosen as it aligns with the size of the smallest sub-quadrant, 32 $\times$ 32 pixels. The resulting uncertainty in CP and MNP regression is illustrated in Fig. 11 alongside the corresponding $3\sigma$ ellipse. Unlike conventional MC scatter plots, this plot reveals distinct clusters due to the integer conversion required for image sub-sampling. Table 7 provides a quantified comparison of the MC analysis results. The navigation performance of the MNP aligns well with the true image center and initial covariance used in the 2500-run MC analysis. The state estimation error is 7.151 pixels for MNP. The CP navigation performance shows a state estimation error of 14.43 pixels. The respective covariance matrices are provided in Table 7.

**Table 7:** Quantitative comparison of navigation performance (MC Run = 2500 samples). The root mean squared error is calculated between the state estimation $(x,y)_{n,pred}$ $(n = 1,\ldots , 2500)$, and the initial image center (200,250).

Approach	MC initial parameters				Post-run MC parameters			
	True *x*	True *y*	${\sigma _x}^2$	${\sigma _y}^2$	$\bar{x}_{pred}$	$\bar{y}_{pred}$	RMSE	Cov(*x*, *y*)
CP	200	250	28.44	28.44	191.7	241.5	14.43	$$\begin{bmatrix} 26.94 & 0.130\\ 0.130 & 64.84 \end{bmatrix}$$
MNP					199.9	246.7	7.151	$$\begin{bmatrix} 45.06 & - 3.485\\ - 3.485 & 11.49 \end{bmatrix}$$

**Fig. 12:**
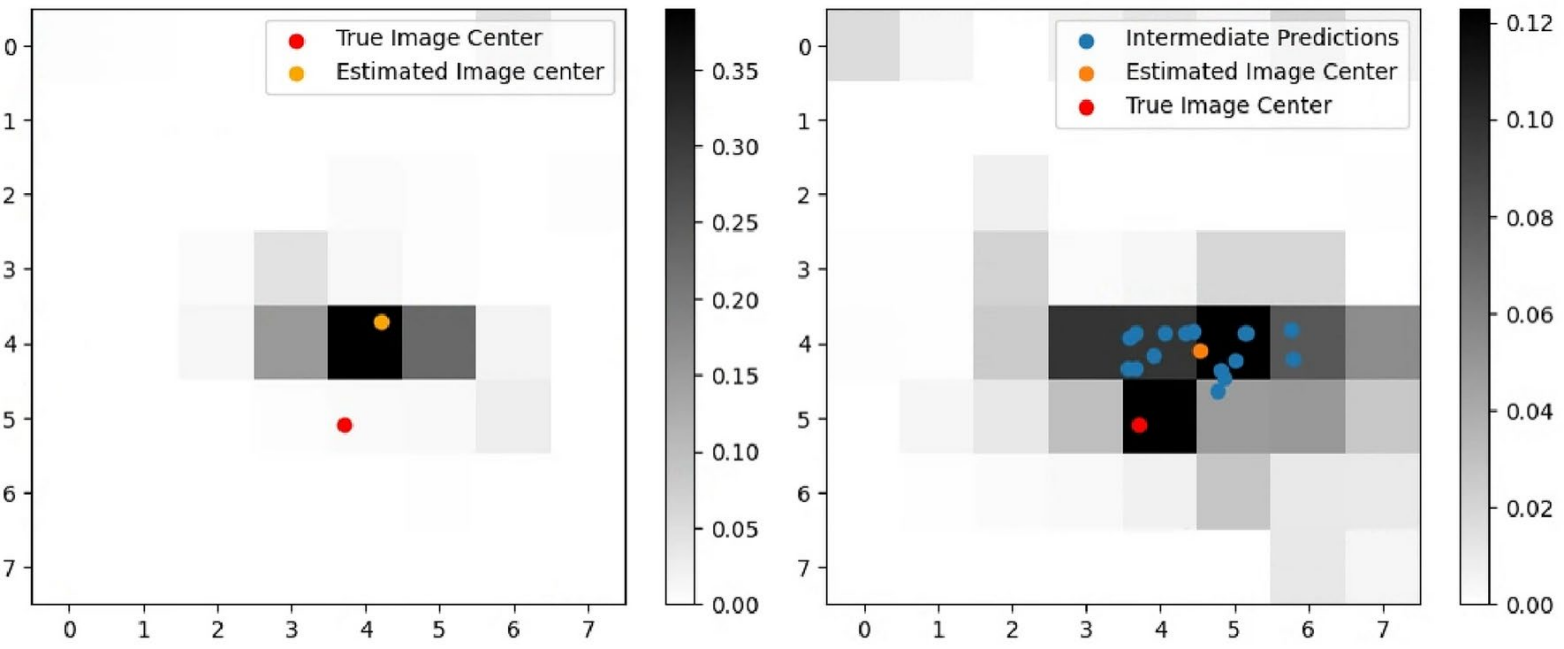
Heatmap of regression results for extreme cases. (Left) Centered prediction result with RMSE of 37.72. (Right) Mean neighborhood prediction result with RMSE of 32.94.

**Fig. 13:**
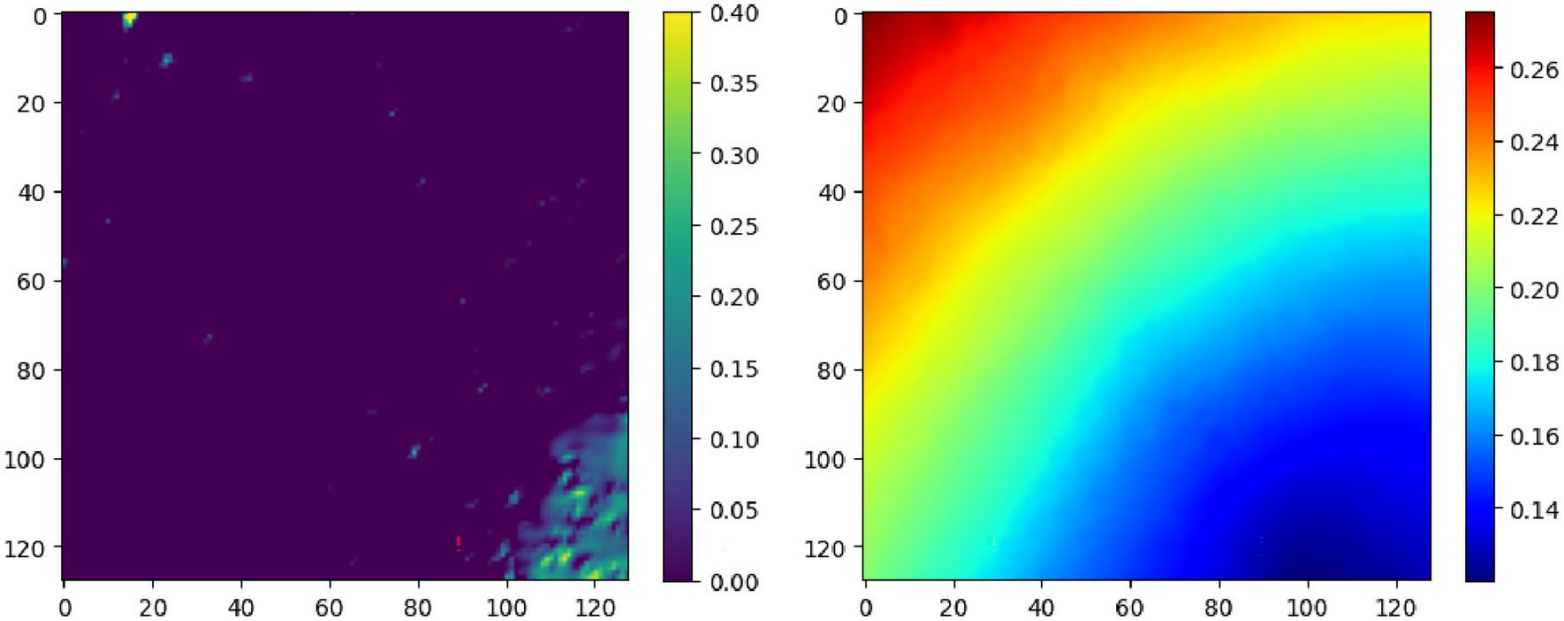
Regression Case 2 with image center at (100,300). (Left) Intensity image. (Right) Depth image.

An additional regression case study was conducted using the extreme conditions. Both the low illumination intensity images and the high degree of error in depth were utilized to prepare the data for regression. To facilitate performance comparison, identical coordinates were employed. The regression results for the extreme case are depicted in Fig. 12. The trained MMCNN struggles to precisely estimate the image center for the extreme case, with RMSE (pixel) of 37.72 for CP and 32.94 for MNP. However, the heatmap facilitates efficient visual interpretation of the performance and trade-offs between the two proposed regression methods. Utilizing neighboring information in MNP allows for a more flexible performance compared to relying solely on information gathered from the sample around the center.

**Fig. 14:**
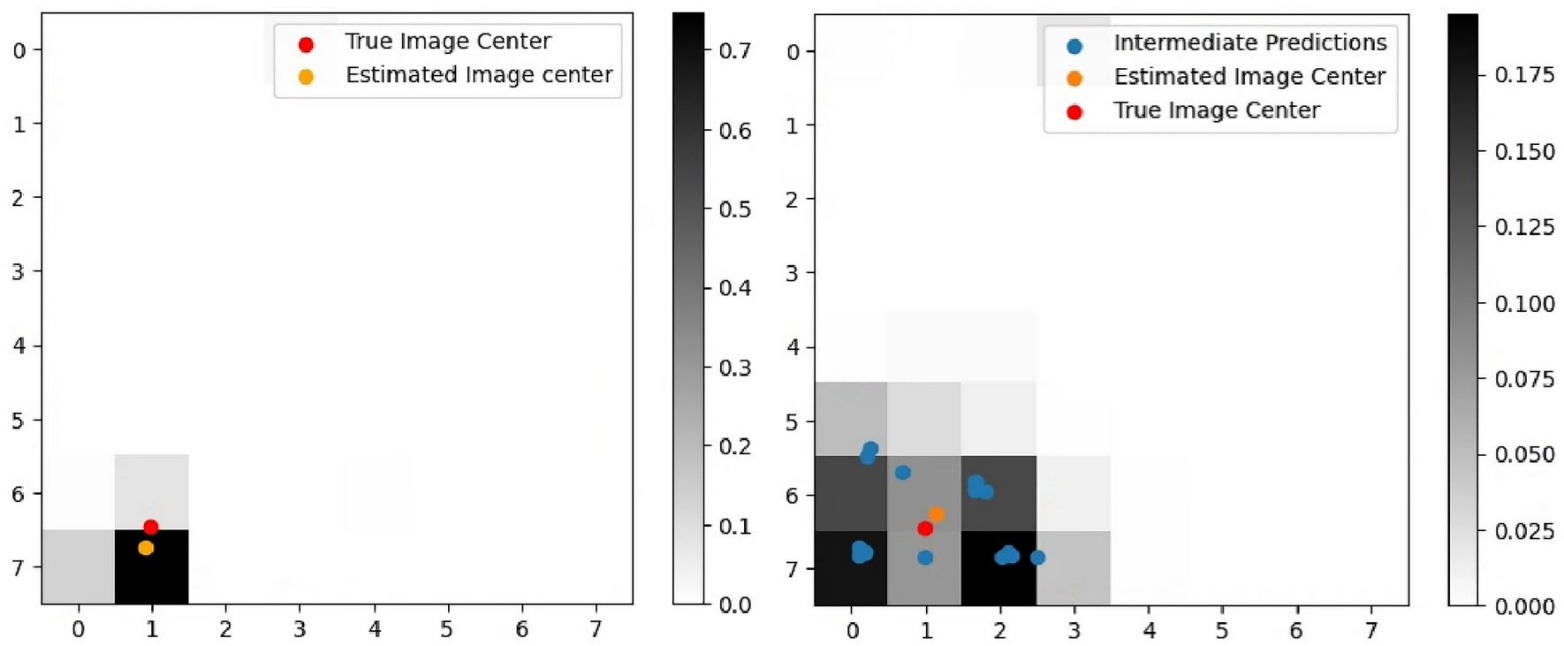
Heatmap of regression results for near-edge scenario. (Left) Centered prediction result with RMSE of 7.78. (Right) Mean neighborhood prediction result with RMSE of 6.68.

An additional regression case study was conducted by placing the image center near the edge of the map with coordinates (*x*, *y*)=(100, 300), where the classification accuracy is the lowest from the intensity mode CNN. Figure 13 shows the sampled images. This case study was undertaken to simulate a scenario where the lunar descent rover deviates significantly away from the center of the map, and low illumination images are captured. Figure 14 shows similar performance with RMSE (pixel) of 7.78 and 6.68 respectively. However, the higher accuracy comes at the computational expense. The computation time is 3.58 s for MNP and 0.266 s for CP, with Intel i7-12700 and NVIDIA GeForce RTX 3070 Ti. Additionally, it is worthwhile to note that as discussed earlier TRN would typically be sought at multiple altitudes and the position estimates would be used to devise a control profile which would guide the lander towards the earmarked landing site ultimately leading to a successful soft landing with enough automated GNC cycles.

## Conclusions

Our study presents a comprehensive investigation into the performance of the Multi-Modal Convolutional Neural Network (MMCNN) in lunar descent TRN tasks under various operating conditions. Promising results in both cascading classification and probabilistic regression tasks were observed, through rigorous test. The integration of multiple modalities, including depth and intensity data, proved advantageous in enhancing the overall accuracy of localization. Notably, extreme scenarios were explored, simulating low illumination conditions and high noise levels in depth data obtained through a stereo camera pair, which are crucial for assessing the robustness of the MMCNN. The reliability of the navigation performance was further validated through Monte Carlo analysis, which demonstrated a state estimation error of 7.151 pixels for Mean Neighborhood Prediction, and 14.43 pixels for Centered Prediction, providing valuable insights into uncertainty quantification. These findings underscore the importance of comprehensive testing in diverse conditions to ensure the reliability and adaptability of localization systems, particularly in real-world lunar exploration missions where perfect conditions cannot always be guaranteed. Further research focusing on refining and evaluating the MMCNN architecture using real-life datasets will be instrumental in advancing lunar surface exploration efforts.

## Data Availability

The Digital Terrain Model used to generate the dataset is available in the Lunar Reconnaissance Orbiter Camera repository, https://wms.lroc.asu.edu/lroc/view_rdr_product/NAC_DTM_APOLLO16. Source codes are available from the corresponding author on request.

## References

[1] Smith, M. *et al.* The artemis program: An overview of NASA’s activities to return humans to the moon. In *2020 IEEE Aerospace Conference*, 1–10 (IEEE, 2020).

[2] Esty, C. C., Lee, D., Martinez, R. & McCarthy, B. Assessment of cislunar staging orbits to support the artemis iii lunar surface mission. In *45th Annual AAS Guidance, Navigation and Control (GN &C) Conference*, AAS 22-762 (2023).

[3] Singh, S., Junkins, J., Anderson, B. & Taheri, E. Eclipse-conscious transfer to lunar gateway using ephemeris-driven terminal coast arcs. *J. Guid. Control. Dyn.* **44**, 1972–1988 (2021).

[4] Singh, S. K., Anderson, B. D., Taheri, E. & Junkins, J. L. Exploiting manifolds of l1 halo orbits for end-to-end earth-moon low-thrust trajectory design. *Acta Astronaut.* **183**, 255–272 (2021).

[5] Singh, S. K., Taheri, E., Woollands, R. & Junkins, J. Mission design for close-range lunar mapping by quasi-frozen orbits. In *70th International Astronautical Congress, Washington DC, USA* (2019).

[6] Trofimov, S., Shirobokov, M., Tselousova, A. & Ovchinnikov, M. Transfers from near-rectilinear halo orbits to low-perilune orbits and the moon’s surface. *Acta Astronaut.* **167**, 260–271 (2020).

[7] Bussey, B., Clarke, S. W., Jenkins, J. & Bailey, B. E. Nasa’s lunar discovery and explorartion program. In *AGU Fall Meeting Abstracts*, vol. 2019, PA54B–11 (2019).

[8] Boazman, S. et al. *Analysis of the Lunar South Polar Region for Prospect, NASA/CLPS* (Tech. Rep, Copernicus Meetings, 2022).

[9] Bhaskaran, S. & Hopkins, J. Astrobotic: Peregrine lunar lander technical program update. In *2017 Annual Meeting of the Lunar Exploration Analysis Group*, vol. 2041, 5019 (2017).

[10] Moon, Q. & Geller, D. K. Batch dilution of precision optimal navigation planning for cislunar environments. *J. Astronaut. Sci.* **70**, 44 (2023).

[11] Epp, C., Robertson, E. & Carson, J. M. Real-time hazard detection and avoidance demonstration for a planetary lander. In *AIAA SPACE 2014 Conference and Exposition*, 4312 (2014).

[12] High Resolution Imaging Science Experiment, HiRISE. hirise.lpl.arizona.edu/.

[13] Zurek, R. W. & Smrekar, S. E. An overview of the mars reconnaissance orbiter (MRO) science mission. *J. Geophys. Res. Planets* **112** (2007).

[14] Downes, L. *et al.* Lunar terrain relative navigation using a convolutional neural network for visual crater detection. In *2020 American Control Conference (ACC)*, 4448–4453 (2020).

[15] Mourikis, A. et al. Vision-aided inertial navigation for spacecraft entry, descent, and landing. *IEEE Trans. Rob.* **25**, 264–280 (2009).

[16] Steiner, T. *et al.* Graph-based terrain relative navigation with optimal landmark database selection. In *2015 IEEE Aerospace Conference* (2015).

[17] Christian, J. et al. Image-based lunar terrain relative navigation without a map: Measurements. *J. Spacecr. Rockets* **58**, 164–181. 10.2514/1.A34875 (2021).

[18] McCabe, J. & DeMars, K. Anonymous feature-based terrain relative navigation. *J. Guid. Control. Dyn.* **43**, 410–421 (2020).

[19] Kim, I. & Singh, S. Bayesian fusion inspired 3d reconstruction via lidar-stereo camera pair. In *International Symposium on Visual Computing*, 299–310 (Springer, 2023).

[20] Maturana, D. & Scherer, S. 3d convolutional neural networks for landing zone detection from lidar. In *Proceedings of (ICRA) International Conference on Robotics and Automation*, 3471 – 3478 (2015).

[21] Liu, B. & Janschek, K. Relative navigation and terrain-based path planning using flash lidar based surfel grid map for asteroid exploration. *IFAC-PapersOnLine* **55**, 85–90. 10.1016/j.ifacol.2023.03.015 (2022) (**(2022). 22nd IFAC Symposium on Automatic Control in Aerospace ACA**).

[22] Adams, D., Peck, C. & Majji, M. Velocimeter light-detection-and-ranging-informed terrain relative navigation. *J. Guid. Control Dyn.* 1–16. 10.2514/1.G007317 (2023).

[23] Sternberg, D. C., Setterfield, T. P., Bailey, E. S., Ansar, A. I. & Johnson, A. E. Demonstration of stereo vision for deorbit descent and landing. vol. 169. Binocular stereo vision system; Flight systems; Landing accuracy; Post processing; Ranging accuracy; Relative navigation; Sensing modalities; Visual odometry, 161–172 (Breckenridge, CO, United states, 2019).

[24] Johnson, A. *et al.**The Lander Vision System for Mars 2020 Entry Descent and Landing*. 2014/46186 (Jet Propulsion Laboratory, National Aeronautics and Space Administration, 2017).

[25] Campbell, T., Furfaro, R., Linares, R. & Gaylor, D. A deep learning approach for optical autonomous planetary relative terrain navigation. *Spacefl. Mech.* **160**, 3293–3302 (2017).

[26] Mancini, P., Cannici, M. & Matteucci, M. Deep learning for asteroids autonomous terrain relative navigation. *Adv. Space Res.* **71**, 3748–3760 (2023).

[27] Scorsoglio, A., Furfaro, R., Linares, R. & Gaudet, B. Image-based deep reinforcement learning for autonomous lunar landing. In *AIAA Scitech 2020 Forum*, 1910 (2020).

[28] Gaudet, B., Linares, R. & Furfaro, R. Adaptive guidance and integrated navigation with reinforcement meta-learning. *Acta Astronaut.* **169**, 180–190 (2020).

[29] Rolen, A. & Singh, S. Autonomous navigation via a cascading cnn framework leveraging synthetic terrain images. In *International Symposium on Visual Computing*, 529–540 (Springer, 2023).

[30] Apollo 16 landing site dtm-digital terrain model (32-bit geotiff). https://wms.lroc.asu.edu/lroc/view_rdr_product/NAC_DTM_APOLLO16.

[31] Bottou, L. Stochastic gradient learning in neural networks. *Proc. Neuro-Nimes* **91**, 12 (1991).

[32] Christian, J. A. Optical navigation using planet’s centroid and apparent diameter in image. *J. Guid. Control. Dyn.* **38**, 192–204 (2015).

